# Quantitative trait loci mapping reveals candidate pathways regulating cell cycle duration in *Plasmodium falciparum*

**DOI:** 10.1186/1471-2164-11-577

**Published:** 2010-10-18

**Authors:** Heather B Reilly Ayala, Mark A Wacker, Geoffrey Siwo, Michael T Ferdig

**Affiliations:** 1 Life Sciences, Bethel College, 1001 Bethel Circle, Mishawaka, IN 46545, USA; 2 Eck Institute for Global Health, Department of Biological Sciences, University of Notre Dame, Notre Dame, IN 46556, USA

## Abstract

**Background:**

Elevated parasite biomass in the human red blood cells can lead to increased malaria morbidity. The genes and mechanisms regulating growth and development of *Plasmodium falciparum *through its erythrocytic cycle are not well understood. We previously showed that strains HB3 and Dd2 diverge in their proliferation rates, and here use quantitative trait loci mapping in 34 progeny from a cross between these parent clones along with integrative bioinformatics to identify genetic loci and candidate genes that control divergences in cell cycle duration.

**Results:**

Genetic mapping of cell cycle duration revealed a four-locus genetic model, including a major genetic effect on chromosome 12, which accounts for 75% of the inherited phenotype variation. These QTL span 165 genes, the majority of which have no predicted function based on homology. We present a method to systematically prioritize candidate genes using the extensive sequence and transcriptional information available for the parent lines. Putative functions were assigned to the prioritized genes based on protein interaction networks and expression eQTL from our earlier study. DNA metabolism or antigenic variation functional categories were enriched among our prioritized candidate genes. Genes were then analyzed to determine if they interact with cyclins or other proteins known to be involved in the regulation of cell cycle.

**Conclusions:**

We show that the divergent proliferation rate between a drug resistant and drug sensitive parent clone is under genetic regulation and is segregating as a complex trait in 34 progeny. We map a major locus along with additional secondary effects, and use the wealth of genome data to identify key candidate genes. Of particular interest are a nucleosome assembly protein (PFL0185c), a Zinc finger transcription factor (PFL0465c) both on chromosome 12 and a ribosomal protein L7Ae-related on chromosome 4 (PFD0960c).

## Background

Malaria is one of the deadliest infectious diseases in the world with the most lethal form, *Plasmodium falciparum*, infecting more than 500 million people each year, two to three million of whom die [1]. The characteristic malarial fevers occur in multiples of 24 hr due to synchronous parasite development and proliferation in the host's red blood cells (RBC), corresponding to cell lysis and massive liberation of new parasites and toxins into the host's bloodstream [2,3]. Clinical studies in South east Asia have demonstrated that parasite lines which proliferate at an increased rate in RBC are more virulent than those with low multiplication rates, indicating a relationship between parasite growth and disease severity [4,5]. The molecular mechanisms directing the rate of parasite growth and development in the erythrocytic cycle are not well understood, underscoring the need to identify candidate genes regulating these processes.

The parasite erythrocytic cycle involves invasion of RBC by a merozoite, followed by a 'ring' stage that begins to ingest haemoglobin. Digestive vesicles merge into a larger digestive vacuole characteristic of the metabolically active trophozoite stage that is active for DNA replication, transcription and translation functions [6-8]. Unlike other eukaryotic organisms, *Plasmodium *spp. does not undergo cytokinesis after each successive round of DNA replication. Instead, DNA replication and mitosis occur multiple times within the same cell body - a process known as endomitosis resulting in the schizont containing 8-32 merozoites [9]. Progression of *P. falciparum *through the erythrocytic cycle takes approximately 48 hours [10]; however, we previously observed a shortened cell cycle in Dd2 compared to HB3 due to a shortened time in the ring and trophozoite stages [11]. These stages correspond to the G1 phase of the cell cycle and make up the majority of the parasites erythrocytic cycle. This observation is consistent with the progression of *Toxoplasma gondii*, another Apicomplexan parasite, through its tachyzoite cell cycle [12].

The development through the erythrocytic cycle requires coordinated expression of distinct sets of genes. Based on expectations of homologous functions with yeast, progression through the malaria parasite cycle is directed by cyclins and cyclin-dependent kinases (CDKs) [13]. Five candidate CDKs have been described in *P. falciparum*; PfPK5 a homologue to CDK1 and CDK5, PfPK6 a homologue to CDK1 and MAPKs, Pfmrk a homologue to CDK7, Pfcrk-1 a homologue to cdc2 [14], and most recently Pfcrk-3 a homologue of CDK-related kinase 3 [15]. Both PfPK5 and Pfmrk have cyclin-dependent activity, whereas PfPK6 does not [16,17]. PfPk5 is active during the erythrocytic cycle and has been implicated in regulation of nuclear division [18,19]. Knock-out studies in *P*. *berghei *with the orthologue for Pfcrk-1 (Pbcrk-1) indicate this cyclin is essential for the completion of the erythrocytic cycle in Plasmodium [20]. Likewise, studies on Pfcrk-3 demonstrate that it is also necessary for the development of the erythrocytic parasite and most likely plays a role in chromatin modification [15]. The Myb-related transcription factors also participate in regulating the expression of genes involved in growth control and cell differentiation [21]. Gissot et al. demonstrated that a decrease in the *P. falciparum *transcription factor PfMyb1 resulted in a 40% parasite growth inhibition altering the transcription of a handful of genes implicated in cell cycle regulation, DNA repair and replication. These included genes such as PfPk5, a proliferating cell nuclear antigen (PCNA) shown to play a role in DNA replication, and a PP1-like phosphatase [22,23]. Additionally, a study by Janse et al. demonstrated that removal of one of two copies of the eukaryotic elongation factor 1α (*eef1α*) from *P. berghei *increased the cell cycle length by extending the G1 phase compared to the parent parasite line [24].

Quantitative trait loci (QTL) mapping combined with the resolution of whole-genome technologies offers unbiased access to genes, biological processes, and mutations throughout a genome and provides a point of entry into the parasite genome from which a list of candidate genes can be refined and narrowed down in an unbiased manner. The goal of QTL mapping is to determine the genetic architecture, i.e. the number, position, and types of genetic contributions, for a given phenotype. Quantitative traits generally involve input from multiple genes acting in ways which may be additive, dominant, epistatic, or interactive and therefore will be associated with multiple QTLs [25,26].

QTL mapping in thirty-four progeny derived from a genetic cross between Dd2, a chloroquine resistant (CQR) parasite from Indochina, and HB3, a chloroquine sensitive (CQS) parasite from Honduras has been used to identify the gene regulating chloroquine (CQ) resistance, *Plasmodium falciparum *chloroquine resistance transporter (*pfcrt*) [27], and more recently the loci involved in quinine (QN) susceptibility [28]. The Dd2 × HB3 cross is characterized by high recombination resolution and cultured haploid progeny lines that can be repeatedly assayed for genome-wide sequence and transcription data, making it an excellent system for identifying genes and mechanisms that have diverged between the parental lines [29,30]. Here we characterize the genetic architecture of erythrocytic cell cycle duration in progeny from the Dd2 × HB3 cross and pinpoint candidate genes regulating this cycle time (CT) in *P. falciparum *[11]. We identify a major QTL on chromosome 12 that interacts epistatically with a locus on chromosome 4 and two additional additive loci to account for nearly 75% of the phenotype variation inherited among the progeny. We draw on existing genome-wide datasets including sequence polymorphisms [31], transcriptional polymorphisms and expression correlations between progeny expression and CT [30,32] and interaction network [33,34] to prioritize candidate genes and their interactions.

## Results

### Progeny inherit quantitative differences in cycle time

**Figure 1:**
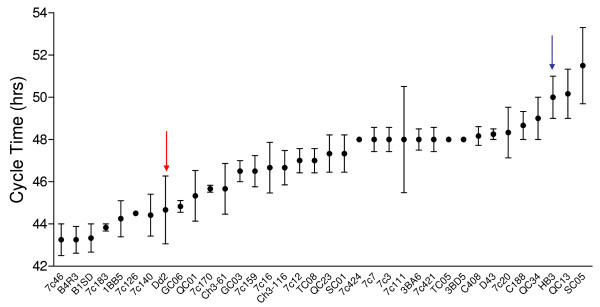
**Cycle Time phenotypes in progeny of Dd2 x HB3 cross**. Progeny are arranged in ascending order of cycle time duration. Dots represent means of a minimum of 3 replicates, error bars are SEM. Parents marked by arrows, Dd2 in red and HB3 in blue.

Previous work identified a statistically significant difference in CT duration in the parent lines (Dd2, 44.1 h; HB3, 49.6 h) [11]. The greatest deviation in cycle time duration was observed in the ring and early trophozoite stages whereas the late trophozoite and schizont stages showed insignificant deviations from each other. Cycle time was measured with biological replication in all 34 progeny of the Dd2 × HB3 cross, revealing a continuous distribution of this trait across the progeny set, indicating that CT is a complex, quantitative trait controlled by multiple genes (Figure 1).

### Quantitative trait loci (QTL) mapping of cycle time

**Figure 2:**
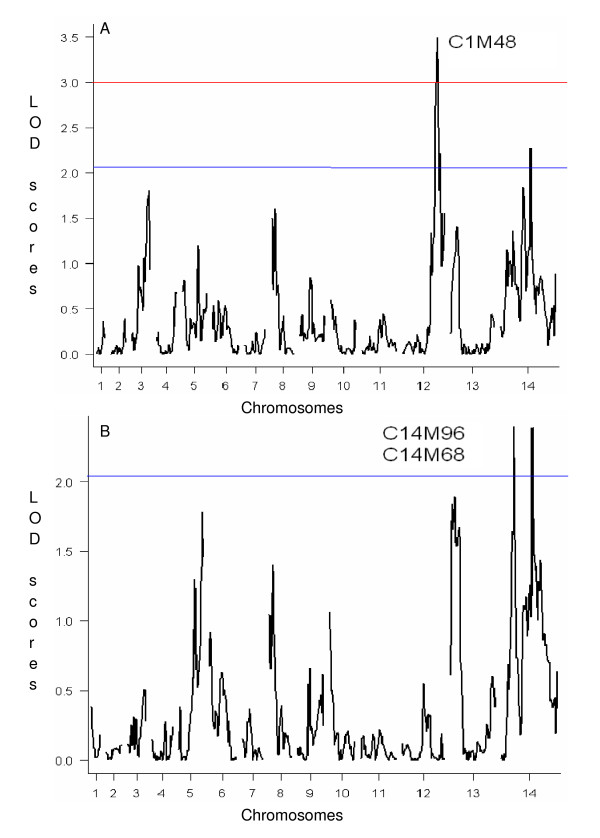
**QTL scans of Cycle Time**. Red line represents significant threshold at 95% and blue line is suggestive at 66% based on 1000 permutations of the data. A) Main scan of cycle time. B) Residual scan of cycle time. Peaks on chromosome 14 from left to right are C14M96 and C14M68.

**Figure 3:**
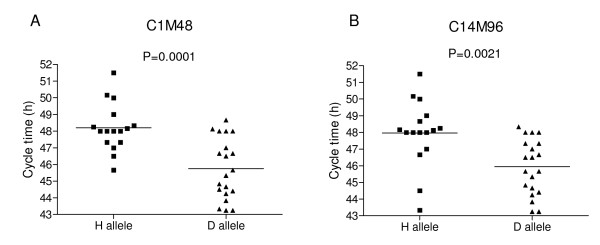
**Allele-specific Cycle Time phenotypes**. A) Marker C1M48 on chromosome 12. The average cycle time for progeny with an H allele or D allele is 48.20 or 45.75 hours respectively. B) Marker C14M96 on chromosome 14. The average cycle time for progeny with an H allele or D allele is 47.96 or 45.94 hours respectively.

**Figure 4:**
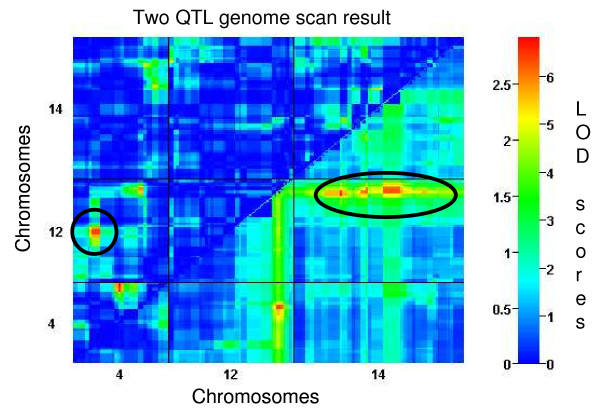
**Residual and interacting Cycle Time QTLs**. Pairwise scan of cycle time based on 34 progeny plus parent lines. Lower right triangle shows additive effects; the circle indicates the additive effect of C1M48 with the chromosome 14 QTLs. The upper left triangle shows the interaction between C1M48 and C4M81. LOD scores are represented by the vertical color bar with the red colors being more significant than the blue colors.

**Figure 5:**
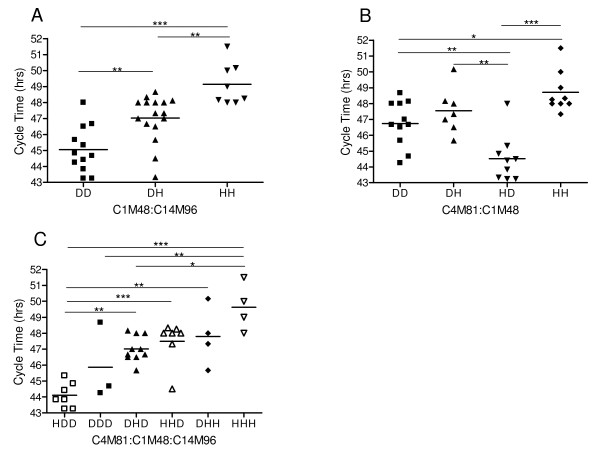
**Cycle Time in progeny inheriting D or H alleles**. D and H represent the parental alleles inherited by the progeny. Lines are the mean cycle time for each allelic haplotypes. A) Progeny inheriting D or H alleles at markers C1M48 and C14M96. The order of these markers is not important. B) Progeny inheriting D and H alleles at markers C4M81 and C1M48, respectively. C) Progeny inheriting H and D alleles for all three markers. The order the markers are listed in is the same order as the indicated haplotypes. * *P *< 0.05, ** *P *< 0.01, *** *P *< 0.001. If no *p *value is stated the difference is not statistically significant.

QTL mapping was used to identify associations between CT and microsatellite (MS) markers distributed throughout the genome to identify chromosome regions carrying polymorphisms that impact CT. QTL scans locate most of the genetic determination of the phenotype variation (43.76%) at one main locus on chromosome 12 at marker C1M48 (Table 1) with a highly significant LOD score (*p *< 0.01 using 1000 permutations) (Figure 2A). In addition to the main effect, several minor peaks on chromosomes 3, 8, 13 and 14 were evident but not significant in the context of the chromosome 12 effect. Consequently, the remaining unassigned fraction of CT variation was analyzed using secondary scans of the residual variation after statistically removing the effect from the chromosome 12 locus; subsequent genome-wide scans identified markers C14M96 and C14M68 on chromosome 14 (LOD scores of 2.388 and 2.392 respectively) (Figure 2B). Inheritance of a D allele at any of these three markers produces progeny with a significantly faster CT than progeny inheriting an H allele (Figure 3A, B). Marker C1M48 exhibits an additive effect with marker C14M96 on chromosome 14 (Figure 4, 5A) such that progeny inheriting a D allele at both markers have a significantly (*P *< 0.0001) faster CT (45.06 hr) than those with an H allele at both markers (49.14 hr) (Figure 5A).

**Table 1:** Summary of QTL effects for Cycle Time

Nearest Marker	Effect	Chr	Position (cM)	LOD	**Sig**.	*df*	SS	Adj. SS	% variance	*F *value	*P *value^a^
C1M48	Main	12	140.1	3.84	99%	1	57.83	63.85	43.76	25.40	4.23E-07
C14M96	Residual	14	124.3	2.39	66%	1	23.84	11.64	7.98	9.26	0.00495
C14M68	Residual	14	50.0	2.39	66%	1	11.78	5.65	3.88	4.50	0.0426
C4M81	Interaction	4	50.0	0.06	n.s.	1	1.13	15.34	10.52	6.10	0.00614
C1M48 x						1	14.85	14.85	10.18	11.82	0.0018
C4M81											
Error						29	36.46				
Total						34	145.89		75.01		

Definitions of terms: Sig. significance values were determined by 1000 permutations of data, *df *- degree of freedom associated with each term in the model, SS - associated sum of squares, Adj. SS - adjusted sums of squares (type III sum of squares adjusting for all other terms in the model), % variance - total variance explained by the adjusted sums of squares, *F *statistics are based on Adj SS, *P *values are based on the *F *distribution.

Next, we ran pairwise scans to search for epistatic (non-additive) interactions between loci; an epistatic interaction is non-additive, such that the allelic contribution of one locus is observable only in the context of allele at the second locus. An interaction between C1M48 and a fourth marker on chromosome 4, C4M81 was identified (Figure 4). No effect was detected for C4M81 independently. Inheritance of the H allele at marker C4M81 and a D allele at C1M48 results in parasites with the fastest CT (44.09 hr) (Figure 5B). Progeny inheriting an H allele at both markers have a significantly (*p *< 0.0001) slower CT (48.71 hr). When all three markers are analyzed together, progeny inheriting the HHH (C4M81, C1M48, C14M96) allele combination have the slowest CT (49.35 hr) while HDD progeny exhibit the fastest CT (44.15 hr) (Figure 5C). Together the multiple QTL model for CT shows four loci, one on chromosome 12, two on chromosome 14 and one on chromosome 4 contributing to 75.01% of the observed variation (Table 1).

### Bioinformatic filtering of CT candidate gene list

**Figure 6:**
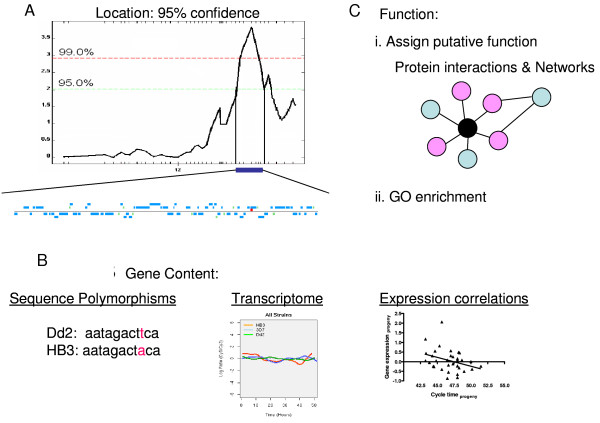
**Constructing and refining a gene list**. A) The gene list is constructed for all genes falling under the peak at 95% confidence using the NCVI database. B) The gene list is refined using whole genome sequence and transcript expression data for Dd2 and HB3 and expression profile correlations with cycle time in the progeny. C) Function is assigned to genes using known functions of interacting genes. The black dot represents the gene of interest, the pink dots are genes with known functions and the blue dots are genes with unknown functions. Genes with an enriched function are maintained in the final list.

The four CT QTLs cover approximately 3.0% of the parasite's 23 Mb genome. A preliminary cumulative list of 165 candidate genes was compiled for these loci (Figure 6A). The comprehensive gene list in these loci, including gene IDs and ontology (GO) annotations, is provided in the supplemental data (See additional file 1). As an initial step, we examine the genes in this locus for functionally relevant candidates that could plausibly be tied to cell cycle regulatory events or DNA replication. Some such candidates include two minichromosome maintenance proteins involved in DNA replication (PFL0560c and PFL0580w) in the chromosome 12 QTL. The chromosome 14 locus contains two serine/threonine protein kinases that could participate in cell cycle control (PF14_0408 and PF14_0392). Marker C14M39 is near a PP1-like protein serine/threonine phosphatase (PF14_0224). The chromosome 4 region contains a Ran binding protein implicated in DNA replication (PFD0950w). Ran proteins are nuclear GTPases thought to be involved in nucleocytoplasmic transport and to play a role in mRNA processing and cell cycle regulation. Notably, genes with functional annotations are in the minority and may not necessarily be involved in the measured phenotype; nearly 68% of the genes encode conserved hypothetical proteins. While functional candidates may merit further interest, we also established unbiased biological criteria to generate comprehensive gene lists irrespective of annotation.

Three criteria were used to refine the gene list: SNP density between parental lines, correlations between transcriptome profiles of the parents, and correlation of the phenotype with gene expression in the progeny (Figure 6B). Given the continued growth of the genome-wide information, e.g. metabolites, protein interactions, chromatin modifications, these filtering criteria will become increasingly expansive in future studies Progeny are likely to inherit structural or expressional polymorphisms in genes which differ between parent lines thereby contributing to observed differences in CT. The top 25% of the values from each criterion was used for cross-filtering. (See additional file 2). SNP density refers to a comparison of the parent lines to determine the number of SNPs (synonymous or nonsynonymous) found within a genomic region. For SNP density the cutoff was set at 1.4 (Additional file 2, Figure s1A). Because not all polymorphic differences are due to structural changes in the gene itself, we also used expression data from the intraerythrocytic developmental cycle. The values used are a correlation of the expression profiles between the parents. In this case the cutoff value was assigned as the lowest 25% of the correlation values at 0.69 (Additional file 2, Figure s1B). Finally, because correlating expression traits with phenotypes can highlight pathways, we evaluated expression profiles for correlation with CT variation within the progeny. The cutoff value was assigned as the top 25% of the expression correlation values at 0.18 (Additional file 2, Figures1C). These three screening criteria were used in parallel each resulting in an approximate 75% reduction of genes. There was some overlap between the genes which were eliminated resulting in 57% reduction in the gene list. Genes meeting any one of these criteria were kept, reducing the list to 71 genes of which 51 are of unknown biological function (71.8%).

To include the large percentage of hypothetical genes in our analytical framework, we incorporated data from protein interactions and gene networks to assign likely functions to hypothetical proteins. Twenty of 71 genes in the candidate list were included in the yeast two hybrid network containing 1267 proteins [33]. In addition, PlasmoMAP, a protein network generated with computational and functional genomics data, provides information for 3667 genes [34]. For our analysis, once a putative function is assigned, genes containing an enriched function are flagged as possibly contributing to CT. Functional enrichment is defined as an over-representation of a particular gene ontology (GO) annotation in a subset of genes whose expression profiles across the progeny correlate with CT. The most enriched GO functions in the genes correlating with CT are: DNA metabolic process (GO: 0006259), antigenic variation (GO: 0020033), protein amino acid dephosphorylation (GO: 0006470) and cell-cell adhesion and cytoadherence to microvasculature (GO: 0016337). Candidate genes involved in any of these functions is given preference as being a gene regulating CT differences in *P. falciparum*. This step effectively eliminated another two thirds of the genes in the initial list of 167 narrowing it down to 24 candidate CT genes (Table 2).

**Table 2:** Final list of prioritized candidate Cycle Time genes

Chr	Gene ID	Gene Name	Proposed function	Data set^a^
12				
	PFL0170w	hypothetical protein	DNA metabolic process	PM
	PFL0185c	nucleosome assembly protein 1, putative	DNA metabolic process	Y2
	PFL0210c	eukaryotic initiation factor 5a, putative	DNA metabolic process	PM
	PFL0250w	hypothetical protein	DNA metabolic process	PM
	PFL0265w	hypothetical protein	DNA metabolic process	PM
	PFL0285w	glyoxalase II family protein, putative	DNA metabolic process	PM
	PFL0295c	hypothetical protein, conserved	antigenic variation	PM
	PFL0315c	hypothetical protein	DNA metabolic process	PM
	PFL0325w	hypothetical protein	antigenic variation	PM
	PFL0435w	hypothetical protein	DNA metabolic process	PM
	PFL0465c	Zinc finger transcription factor (krox1)	DNA metabolic process	Y2
	PFL0500w	50S ribosomal protein L1, putative	DNA metabolic process	Y2, PM
	PFL0575w	hypothetical protein	antigenic variation	Y2
	PFL0585w	PfpUB Plasmodium falciparum polyubiquitin	DNA metabolic process	Y2, PM
	PFL0630w	iron-sulfur subunit of succinate dehydrogenase	DNA metabolic process	PM
14-1				
	PF14_0217	hypothetical protein	antigenic variation	PM
	PF14_0218	actin, putative	antigenic variation	PM
14-2				
	PF14_0385	hypothetical protein	antigenic variation	PM
	PF14_0396	hypothetical protein	DNA metabolic process	PM
	PF14_0397	hypothetical protein, conserved	DNA metabolic process	PM
	PF14_0404	hypothetical protein	antigenic variation	PM
	PF14_0407	hypothetical protein	DNA metabolic process	PM
4				
	PFD0950w	ran binding protein 1	DNA metabolic process	GO
	PFD0960c	ribosomal protein L7Ae-related protein, putative	DNA metabolic process	PM

^a^Data set refers to the interaction data used to imply the function of the protein. Data from PlasmoMAP is denoted by PM and data from the yeast two-hybrid is denoted by Y2. Genes with a known function from the gene ontology are indicated by GO.

**Figure 7:**
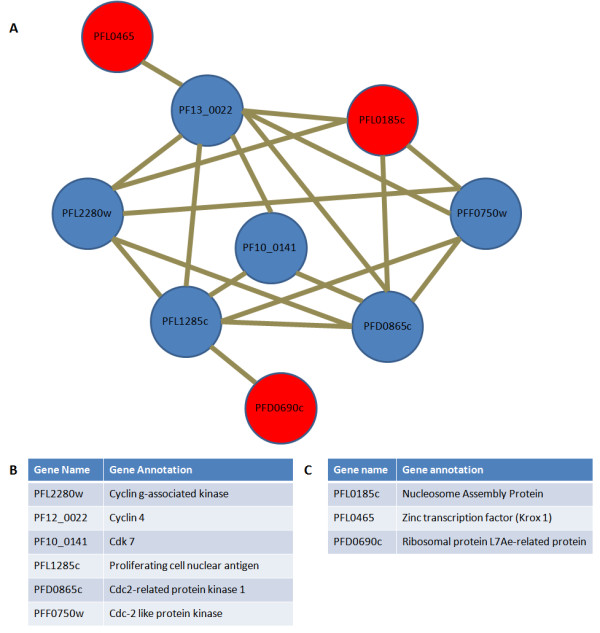
**Putative interactions based on domains of genes within the QTL on chromosome 12 and 4**. A) An interaction network of proteins found to interact with cyclins. Seeded proteins are shown in blue; proteins from the QTL are shown in red. Proteins are identified by their gene name. Only proteins from the final candidate gene list were tested for interactions. B) Gene name and annotation for the proteins used to seed the interaction network. Genes were selected based on their involvement will cell cycle progression. C) Gene name and annotation of proteins from the final candidate list found to have interaction with the seeded proteins in the network.

Given the well-described role of cyclins in regulating cell cycle progression, we ascertained the potential direct physical interactions with cyclins among our candidate genes. Using domain information for proteins encoded by candidate genes within the QTL, we built an interaction network between the proteins using cyclins as a seed using a previously described algorithm for predicting domain-domain interactions given proteins with identifiable domains [35]. Only genes annotated as coding for cyclins were considered (Figure 7). A protein-protein interaction network in which three of the genes from the list of 24 candidates interact with the seeded proteins was produced by this procedure (Figure 7).

We extended this network to test possible interactions involving the additional 104 genes from the initial candidate gene list, but no additional interactions were found. Several proteins are connected to the network, including nucleosome assembly protein (PFL0185c) and Zinc transcription factor Krox 1 (PFL0465), both found at the main QTL on chromosome 12, and the protein ribosomal protein 7LAe-related protein (PFD0960c) located at the interacting locus on chromosome 4. The nucleosome assembly protein (PFL0185c) was selected based on its relatively weak correlation between Dd2 and HB3 transcription profiles, however, SNP density and phenotype-expression correlation highlight differences. In contrast, the zinc finger transcription factor (PFL0465c) had a high SNP density between Dd2 and HB3 exhibiting 3 nonsynonymous and 2 synonymous SNPs. In addition to having a high SNP density, the ribosomal protein also showed a strong correlation between the cycle time phenotype and expression data across the progeny.

## Discussion

We showed previously that the CQR and multi-drug resistant parasite, Dd2, proliferates faster than its CQS counterpart, HB3, and that one component of this faster growth rate is due to a shorter G1 phase in the erythrocytic cycle of Dd2 [11]. Here we demonstrate that progeny inherit CT as a range of values indicative of a complex trait influenced by multiple genes. Our multi-locus genetic model of the determinants of this trait includes four loci accounting for 75.01% of the variance in CT; the main effect on chromosome 12, two additive interactions on chromosome 14, and a locus on chromosome 4 that interacts epistatically with the large chromosome 12 effect (Table 1). Progeny inheriting a D allele at the markers on chromosomes 12 and 14 exhibit significantly faster CTs compared to progeny inheriting an H allele (Figure 3).

This difference in CT length between parasites appears to be a result of a shortened or elongated amount of time in the ring and trophozoite stages [11,24]. It is during these stages that the DNA replication, transcription and translation occurs [6,7]. To narrow the list of genes falling within QTL regions, we developed a method to draw on the existing databases to prioritize candidate genes using genome-wide data from independent sources and to indicate functions of candidates based on their relationship with other known genes, even in the absence of an annotation.

Plasmodium spp. have unique features of cell cycle regulation; consequently standard homology-based assumptions may miss important components. By utilizing an unbiased approach that relies on both genetics and networks of biological interactions we can retain hypothetical genes in our model, while also drawing on the rich data available from model organisms. Interestingly, all three of the genes predicted by our protein interaction network were also contained in the final gene list produced by the unbiased filtering (Figure 7, Table 2).

Functional analysis of genes correlating with the CT trait identified an enrichment of genes involved in DNA metabolism (GO:0006259). According to the gene ontology database http://amigo.geneontology.org/cgi-bin/amigo/go.cgi, the GO annotation of DNA metabolism includes a suite of molecular functions including: DNA replication, elongation, ligation, maintenance, mutagenesis, repair, modification and packaging. Mutations within genes involved in the DNA metabolic process would affect the efficiency of DNA replication and therefore the rate at which it is completed. Assuming *P. falciparum*, like other eukaryotic organisms, has a DNA checkpoint in its cell cycle, a delay in DNA replication would have an effect on the progression of the parasite through its erythrocytic cycle.

Studies in yeast and bacteria have shown that progression through the cell cycle is regulated by a complex process of protein synthesis, degradation and phosphorylation using cyclins, cyclin-dependent kinases (CDKs) and ubiquitin. These proteins ensure that imperative steps are completed before the cell moves into the next stage of the cell cycle. For example, cells must first complete DNA replication before moving into mitosis thereby preventing the formation of new cells which do not contain the proper number of DNA molecules. Several homologues for cyclins and CDKs have been identified in the Plasmodium genome; although their specific roles are poorly understood [13]. No known Plasmodium cyclins and CDKs were found in our QTL, indicating that these genes (cyclins and CDKs) are not involved in this phenotype; however, our method allows us to integrate candidates with possible role for these classic cell cycle determinants. This approach is based on the hypothesis that genes underlying QTL effects operate indirectly through physical interactions with key cell cycle regulators, specifically cyclins. The predicted domain-domain based protein interaction network identified interactions between three genes in the candidate list with cyclins, depicting the possibility that the genes could regulate the cell cycle indirectly by relaying regulatory messages. This form of indirect regulation of biological processes such as the cell cycle could enable biological systems to respond to different environmental stimuli to which they have no direct interactions with.

We highlight candidate genes in the chromosome 12 QTL, and one at the interacting loci on chromosome 4, that could play a role in DNA metabolism and have an effect on the length of the parasite's erythrocytic cycle: PFL0185c, nucleosome assembly protein and PFL0465c, Zinc finger transcription factor (Krox1) on chromosome 12 and PFD0960c ribosomal protein L7Ae-related protein on chromosome 4. All of these were identified as candidates by the protein interaction network. Nucleosome assembly proteins (NAPs) are involved in chromatin assembly and remodelling in order to allow for replication, transcription, recombination and repair of DNA. They play a role in the global changes of chromatin structure which control the progression of *P. falciparum *through its lifecycle [36]. A recent study characterized the role of two nucleosome assembly proteins in *P. falciparum*, PfNapS (PFI0930c) and PfNapL (PFL0185c), which bind to histones; phosphorylation of these proteins by casein kinase II results in conformational changes increasing the histone binding affinity [37]. The PfNapS was shown to have a higher affinity for histones than PfNapL and is proposed to strip the histones from PfNapL. It shuttles the histones into the nucleus and deposits them onto DNA for chromatin remodelling. A polymorphism in this gene could affect the function of PfNapL and could affect the rate at which replication occurs resulting in a change of cell cycle length. PfNapL localizes in the cytoplasm of the parasite. Attempts to disrupt the gene were not successful suggesting that its role in *P. falciparum *is essential for parasite survival [38].

The zinc transcription factor (Krox1) is also candidate for regulating differences in cycle duration. Krox-1 was identified in sea urchin embryos and shown to play a role in the development of the vegetal-plate [39]. It is in the same family as the Blimp-1 transcription factor which is required for the development of plasma cells from B cells [40,41]. Blimp-1 has been shown to be up-regulated in malignant cells compared to B-cells and is involved in the transduction pathway for growth factors in multiple myeloma [42]. A recent study has shown that Krox-1 is unregulated in *P, falciparum *parasites treated with inhibitors of s-adenosylmethinonine decarboxylase/ornithine decarboxylase which block the synthesis of spermidine and subsequently cell cycle progression [43].

The third candidate identified by the protein interaction network is ribosomal protein L7Ae-related protein, PFD0960c. The gene for this protein is located at the interacting loci on chromosome 4. The network shows a path through which this gene could have an epistatic effect, on the genes located on chromosome 12. A study of larynx carcinoma cells resistant to the drug taxol, which stalls cells in the G2/M phase of the cell cycle, were shown to have elevated levels of ribosomal protein L7a [44]. Expression data between the parents shows the multi-drug resistant Dd2 to have an approximate 3.7-fold increase of PFD0960c compared to HB3, suggesting the malarial ribosomal protein L7Ae may play an important role in regulation of the parasite lifecycle [30].

## Conclusions

Overall, this study provides a new method to elucidate genes and functions involved in the regulation of the *P. falciparum *cell cycle. Regulation of the parasite erythrocytic cycle is complex, involving multiple genes and pathways; consequently, a single genetic cross will capture only a subset of the genetic components existing in the general population. The genes and interactions identified in our analysis point to previously unrecognized mechanisms underlying divergent CT between HB3 and Dd2. We also describe an unbiased method to integrate QTL candidates with the wealth of published data to narrow candidate gene lists. Using a protein interaction network seeded with cell cycle regulators, we found direct contact between 3 positional candidate genes and classical cell cycle genes. The overlap between these methods indicates that systematic, unbiased filtering can reveal new interactions and functions associate with key phenotypes.

## Methods

### Parasite culture

Parasites were grown in complete media (CM) [370 μM hypoxanthine (Hx) (Sigma), and 25mM HEPES (Sigma); 0.5% Albumax II (Gibco), 10 μg/mL gentamycin (Gibco), and 0.225% sodium bicarbonate (Biosource) in RPMI-1640 medium (Gibco) at approximately 3.5% hematocrit (hct) in O^+ ^RBC. Cultures were maintained at a constant pH, 7.0-7.5, temperature, 37°C, and atmosphere, 5% CO_2_/5% O_2_/90% N_2 _with daily media changes. Brief descriptions of previously used growth assays are given below [11].

### Determination of cycle time

Cultures were tightly synchronized by two treatments of D-sorbitol on consecutive days and resuspended in 5 mL of CM at 1% parasitemia. Experiments were initiated with parasites in the schizont stage immediately following the second synchronization. Segmenting schizonts provide a visually definitive, narrow morphological window (approximately 4hr) for precise stage identification. Progression of the parasite culture throughout the erythrocytic cycle was monitored using Giemsa-stained smears. The time at which the culture had the highest percentage of segmenting schizonts was defined as the schizont peak. A small volume smear (approximately 50 μl) from each culture was made every 2 hr, originating from the same culture for each biological replicate, throughout the 12 hr window spanning the schizont peak for four consecutive life cycles. CT is defined as the duration between schizont peaks. Average CT was calculated from 3-4 consecutive life cycles and used in QTL analysis.

### QTL Analysis

Statistical analysis of the QTL data was carried out by computational approaches previously described [45]. The erythrocyte stages of the parasites used for genotyping and phenotyping are haploid; therefore, only two genetic classes are present for each locus, and the computational approach is equivalent to an analysis of recombinant inbred lines. Regression analysis and QTL mapping was done using R 2.4.1 software available through Jackson Laboratories http://pga.jax.org/qtl/download.html. Interval mapping was carried out at the average resolution (5 cM) of the *P. falciparum *genetic linkage map which contains 819 microsatellite markers [29]. One thousand permutations of the trait values determined 10%, 5%, and 1% thresholds [46]. Main QTL and corresponding mean trait values from the primary scans were used to obtain estimates of residual empirical thresholds to search for additional QTL via secondary scans [46]. This secondary scan procedure was equivalent to installing the a priori QTL as covariates, thereby attributing a large portion of the overall variation to these QTL and allowing for detection of additional loci controlling the remaining variation. Pairwise scans were performed at 5 cM spacing, testing all possible pairs of QTL locations for association with the trait. The likelihood calculated for a pair (one main locus and an interacting locus) was compared with that of the null model (no pairwise effects). Significance was assessed by permutation analysis of every pairwise combination of marker alleles. Where an interaction was identified, a sequential series of statistical tests was performed to distinguish a true epistatic interaction from additive effects (independent gene actions) and hitchhiking (false positives resulting from related genotypes). These subsequent tests used a stringent nominal (comparison-wise) cut-off (*P = *0.01). For the final model an ANOVA was conducted by incorporating all the main and interacting QTL identified by the main, secondary and pairwise scans into a multiple regression analysis. Contributions of a QTL (or interaction) in combination with all other QTL was determined by calculating *F*-statistics from adjusted (type III) sums of squares. For this regression analysis, inheritance of the Dd2 parent allele was coded as +0.5 and inheritance of the HB3 parent allele was coded as -0.5. Accordingly, the main effect estimate for any locus was taken to be the difference between the effect of the Dd2 genotype and the HB3 genotype. Each interaction parameter was computed from the different effects on a main QTL of its paired Dd2- or HB3-type interacting QTL.

### Constructing a candidate gene list

The candidate gene list was constructed, refined and prioritized using three selection criteria: 1) position, 2) structural and expressional polymorphisms and 3) functional analysis. Any genes falling between markers flanking the 95% confidence range of the QTL peak were included in a preliminary list of candidate genes along with gene IDs, gene names and approximate gene positions (kb) taken from the NCBI database http://www.ncbi.nlm.nih.gov.

Structural (SNPs in the ORF) polymorphisms, expressional polymorphisms between Dd2 and HB3 and correlations between transcription and CT phenotype in the progeny were used to refine the list of candidate genes. Genes that satisfied one of these criteria were retained. The top 25% percentile of the genes (or in the case of the transcription data, the bottom 25%) was kept (Additional file 2, Figure s1). The SNP position, type (non-coding, nonsense, synonymous, non-synonymous), and coding sequence SNP density were obtained from PlasmoDB v5.2 http://plasmodb.org/plasmo/ and recorded for every gene in the preliminary list. The SNP density refers to the number of SNPs in that particular region of the genome. For the initial 165 CT candidate genes, the SNP data was confirmed by comparing Dd2 and HB3 consensus sequences obtained from the Broad Institute (provided by Sarah Volkman). Genes containing multiple SNPs, defined as any gene with a SNP density greater than 1.4 (this was the 25 percentile cutoff), were placed in a refined list of candidate genes. Secondly, expressional polymorphisms were analyzed using correlation values between the Dd2 and HB3 transcriptional profiles downloaded from the DeRisi Lab Malaria Transcriptome Database http://malaria.ucsf.edu/comparison/index.php[47]. If a gene had more than one correlation, the values were averaged and an absolute value was used. All correlation values fell between 0 and 1. Genes with an absolute value correlation less than 0.69 (the 25 percentile cutoff) between the parent lines was maintained in the refined list of genes. Finally, correlation coefficients were calculated between the CT phenotype and gene expression at 18 hr post invasion across the progeny. The expression data from 35 cDNA arrays for 7665 probes representing 5150 ORFs, one for each progeny parasite, at 18 hr post invasion was obtained from Joe Gonzales [30]. A correlation coefficient ≥ 0.18 were retained (25 percentile cutoff).

The third criterion, functional analysis, is composed of two steps: 1) defining gene enrichment and 2) assigning a function to genes in the refined list. Gene enrichment is determined as follows: the CT phenotype was compared with 18 hr post-invasion gene expression in the progeny to calculate a correlation coefficient [30]. Of the 5150 ORFs represented, and with a correlation coefficient, ≥ .0.30 was selected and the corresponding gene was used to determine enrichment. The gene ontology (GO) annotation for each selected gene was obtained from PlasmoDB v5.2. The GO annotations were consolidated under broad headings and screened for enrichment within the CT genes. Gene enrichment was calculated using a hypergeometric distribution [32]. Genes within the refined list were assigned a function first based on their GO term taken from PlasmoDB. Hypothetical genes lacking a function were given a putative function using interaction data from yeast two hybrids (provided on PlasmoDB) [33] and PlasmoMAP http://www.cbil.upenn.edu/plasmoMAP/index-v1.html[34]. Genes with a term associated with GO in the enriched set were retained. The filtered gene list represents genes falling within the confidence range of the QTL, contain polymorphisms between the parent lines, and bioinformatic evidence of a functional relationship to CT.

### Protein interaction network

A protein interaction network was constructed between candidate genes and cyclins, using the software Cytoprophet [35], based on physical interactions between known protein domains in the Pfam domain database, database of interacting proteins [48] and protein databank [49]. In order to determine whether the candidate genes may mediate effects on the cell cycle, the network was seeded with *P. falciparum *cyclin related proteins that play a key role in cell cycle regulation and have domains identifiable in the Pfam database. These proteins are cyclin g associated kinase (PFL2280w), cyclin 4 (PF13_0022), putative cdk 7 (PF10_0141), putative proliferating cell nuclear antigen, PCNA (PFL1285c), cdc2-related protein kinase 1 (PFD0865c), and cyclin dependent protein kinase (PFF0750w).

## Authors' contributions

HBA collected the CT data for all the progeny, performed the QTL analysis, and constructed the candidate gene list. MAW and GS constructed the protein interaction network and performed the corresponding analysis. MTF coordinated the study and helped to draft the manuscript. All authors contributed to writing the manuscript. All authors read and approved the final manuscript.

## Supplementary Material

Additional file 1 Table with full list of genes from four Cycle Time QTLs Click here for file

Additional file 2 **Scatter plots of criteria used to refine gene list** The x-axis represents the 104 genes in the preliminary gene list. They are arranged in ascending order for each of the three criteria used. A) SNP density between HB3 and Dd2 taken from PlasmoDB. B) Correlations of transcriptome for each gene between HB3 and Dd2 [47] C) Correlation of gene expression at 18 hours post-invasion between HB3 and Dd2 [30]. Click here for file
