# Supplementary material for: Quantitative trait loci mapping reveals candidate pathways regulating cell cycle duration in Plasmodium falciparum
Source: BMC Genomics. 2010 Oct 18;11:577. doi: 10.1186/1471-2164-11-577 (PMC3091725; doi:10.1186/1471-2164-11-577)
Supplement: Additional file 1 — Table with full list of genes from four Cycle Time QTLs [file 1471-2164-11-577-S1.PDF]

Additional file 1: Full gene list for four cycle time QTLs

Chromosome 12 Main Effect

Gene ID	*Gene Name*
PFL0155c	hypothetical protein
PFL0160w	hypothetical protein
PFL0165c	hypothetical protein
PFL0170w	hypothetical protein
PFL0175c	hypothetical protein, conserved
PFL0180w	cytochrome c1 heme lyase, putative
PFL0185c	nucleosome assembly protein 1, putative
PFL0190w	ubiquitin-conjugating enzyme e2, putative
PFL0195w	hypothetical protein
PFL0200c	hypothetical protein
PFL0205w	hypothetical protein
PFL0210c	eukaryotic initiation factor 5a, putative
PFL0215c	hypothetical protein
PFL0220c	hypothetical protein
PFL0225c	hypothetical protein
PFL0230w	hypothetical protein
PFL0235w	hypothetical protein, conserved
PFL0240w	hypothetical protein
PFL0245w	hypothetical protein
PFL0250w	hypothetical protein
PFL0255c	uga suppressor tRNA-associated antigenic protein, putative
PFL0260c	hypothetical protein
PFL0265w	hypothetical protein
PFL0270c	hypothetical protein
PFL0275w	hypothetical protein
PFL0280c	hypothetical protein
PFL0285w	glyoxalase II family protein, putative
PFL0290w	hypothetical protein
PFL0295c	hypothetical protein, conserved
PFL0300c	phosphoesterase, putative
PFL0305c	hypothetical protein
PFL0310c	eukaryotic translation initiation factor 3 subunit 8, putative
PFL0315c	hypothetical protein
PFL0320w	hypothetical protein
PFL0325w	hypothetical protein
PFL0330c	DNA-directed RNA polymerase III subunit, putative
PFL0335c	eukaryotic translation initiation factor 5, putative
PFL0340w	hypothetical protein
PFL0345w	hypothetical protein
PFL0350c	hypothetical protein
PFL0355c	hypothetical protein
PFL0360c	hypothetical protein
PFL0365c	hypothetical protein
PFL0370w	hypothetical protein
PFL0375w	hypothetical protein
PFL0380c	tRNA delta(2)-isopentenylpyrophosphate transferase, putative

PFL0385c	blood stage antigen 41-3 precursor
PFL0390c	hypothetical protein
PFL0395c	hypothetical protein
PFL0400w	50S ribosomal protein L29, putative
PFL0405w	hypothetical protein
PFL0410w	hypothetical protein
PFL0415w	acyl carrier protein, mitochondrial precursor, putative
PFL0420w	hypothetical protein
PFL0425c	hypothetical protein
PFL0430w	tim10 homolog, putative
PFL0435w	hypothetical protein
PFL0440c	hypothetical protein
PFL0445w	hypothetical protein
PFL0450c	hypothetical protein
PFL0455c	hypothetical protein
PFL0460w	u6 snRNA-associated Sm-like protein, putative
PFL0465c	Zinc finger transcription factor (krox1)
PFL0475w	3',5'-cyclic-nucleotide phosphodiesterase, putative
PFL0470w	hypothetical protein
PFL0480w	porphobilinogen deaminase, putative
PFL0485w	hypothetical protein
PFL0490c	hypothetical protein
PFL0495c	ABC transporter, putative
PFL0500w	50S ribosomal protein L1, putative
PFL0505c	hypothetical protein
PFL0510c	hypothetical protein
PFL0515w	hypothetical protein
PFL0520c	hypothetical protein
PFL0525w	hypothetical protein
PFL0530c	hypothetical protein
PFL0535c	hypothetical protein
PFL0540w	mannosyltransferase, putative
**C1M48**	***TIP OF THE PEAK***
PFL0545w	kinesin-related protein, putative
PFL0550w	hypothetical protein
PFL0555c	hypothetical protein
PFL0560c	minichromosome maintenance protein, putative
PFL0565w	heat shock protein DNAJ homolog Pfj4
PFL0570c	hypothetical protein
PFL0575w	hypothetical protein
PFL0580w	DNA replication licensing factor mcm5, putative
PFL0585w	PfpUB Plasmodium falciparum polyubiquitin
PFL0590c	p-type ATPase, putative
PFL0595c	glutathione peroxidase
PFL0600w	hypothetical protein
PFL0605c	hypothetical protein
PFL0610w	hypothetical protein
PFL0615w	hypothetical protein
PFL0620c	choline transporter
PFL0625c	eukaryotic translation initiation factor 3 subunit 10, putative

PFL0630w	iron-sulfur subunit of succinate dehydrogenase
PFL0635c	bromodomain protein, putative
PFL0640w	hypothetical protein
PFL0645c	hypothetical protein
PFL0650c	hypothetical protein
PFL0655w	hypothetical protein
PFL0660w	dynein light chain 1, putative
PFL0665c	RNA polymerase subunit 8c, putative
PFL0670c	Bi-functional aminoacyl-tRNA synthetase, putative

Chromosome 14 Secondary effect

Gene ID	*Gene Name*
**QTL 1**	
PF14_0382	metalloendopeptidase, putative
PF14_0383	hypothetical protein
PF14_0384	hypothetical protein
PF14_0385	hypothetical protein
PF14_0386	hypothetical protein
PF14_0387	hypothetical protein
PF14_0388	hypothetical protein
PF14_0389	hypothetical protein
PF14_0390	hypothetical protein
PF14_0391	ribosomal protein L1, putative
PF14_0392	Ser/Thr protein kinase, putative
PF14_0393	structure specific recognition protein, putative
PF14_0394	hypothetical protein
PF14_0395	hypothetical protein
PF14_0396	hypothetical protein
PF14_0397	hypothetical protein, conserved
PF14_0398	hypothetical protein
PF14_0399	ADP-ribosylation-like factor, putative
PF14_0400	hypothetical protein, conserved
PF14_0401	hypothetical protein
PF14_0402	hypothetical protein
PF14_0403	protein prenyltransferase alpha subunit, putative
PF14_0404	hypothetical protein
PF14_0405	hypothetical protein
PF14_0406	hypothetical protein
PF14_0407	hypothetical protein
PF14_0408	Ser/Thr protein kinase, putative
PF14_0409	hypothetical protein
PF14_0410	hypothetical protein
PF14_0411	small nuclear ribonuclear protein, putative
PF14_0412	hypothetical protein
PF14_0413	hypothetical protein
PF14_0414	hypothetical protein, conserved
PF14_0415	dephospho-CoA kinase, putative
PF14_0416	hypothetical protein
PF14_0417	heat shock protein, putative
PF14_0418	hypothetical protein

PF14_0419	hypothetical protein
PF14_0420	hypothetical protein
PF14_0421	hypothetical protein, conserved
PF14_0422	hypothetical protein

QTL 2

PF14_0215	hypothetical protein
PF14_0216	hypothetical protein
PF14_0217	hypothetical protein
PF14_0218	actin, putative
PF14_0219	hypothetical protein
PF14_0220	hypothetical protein
PF14_0221	hypothetical protein, conserved
PF14_0222	hypothetical protein
PF14_0223	cyclophilin, putative
PF14_0224	PP1-like protein serine/threonine phosphatase
PF14_0225	hypothetical protein
PF14_0226	hypothetical protein
PF14_0227	calcium-dependent protein kinase, putative
PF14_0385	hypothetical protein

Chromosome 4 Interacting Loci

Gene ID	*Gene Name*
PFD0935c	hypothetical protein
PFD0940w	hypothetical protein
PFD0945c	hypothetical protein
PFD0950w	ran binding protein 1
PFD0955w	hypothetical protein
PFD0960c	ribosomal protein L7Ae-related protein, putative
PFD0970c	hypothetical protein
