# Supplementary figures and images for: Quantitative trait loci mapping reveals candidate pathways regulating cell cycle duration in Plasmodium falciparum
Source: BMC Genomics. 2010 Oct 18;11:577. doi: 10.1186/1471-2164-11-577 (PMC3091725; doi:10.1186/1471-2164-11-577)

A

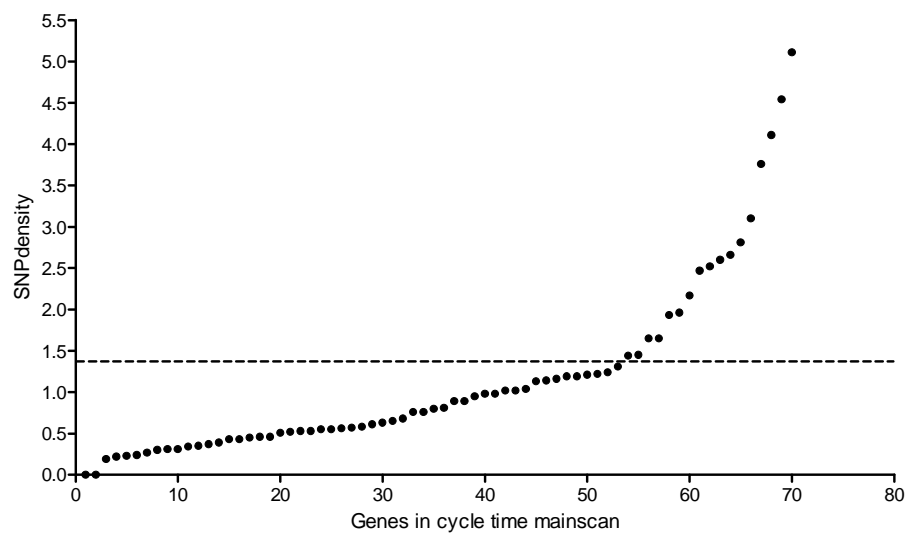

B

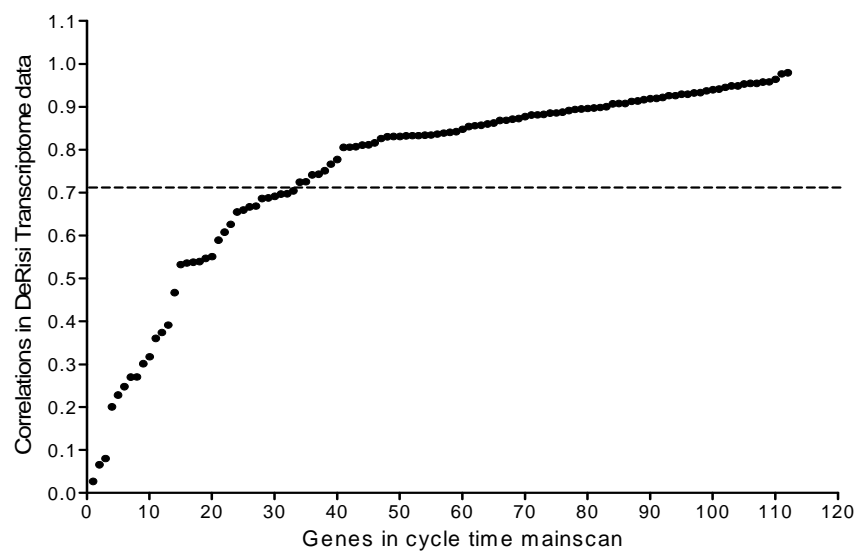

C

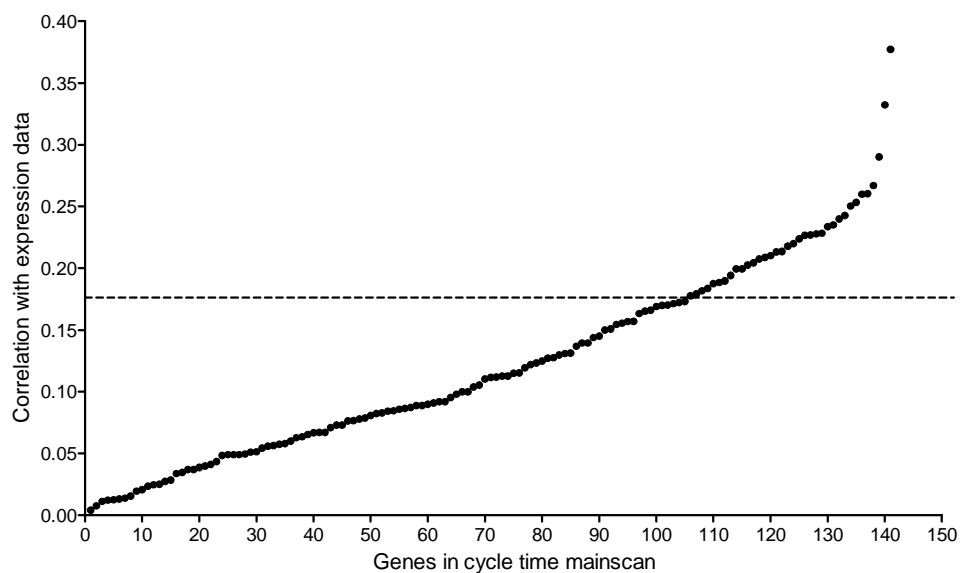

Supplement: Additional file 2 — Scatter plots of criteria used to refine gene list The x-axis represents the 104 genes in the preliminary gene list. They are arranged in ascending order for each of the three criteria used. A) SNP density between HB3 and Dd2 taken from PlasmoDB. B) Correlations of transcriptome for each gene between HB3 and Dd2 [47] C) Correlation of gene expression at 18 hours post-invasion between HB3 and Dd2 [30]. [file 1471-2164-11-577-S2.PDF]
